# Considerations for Choosing Soluble Immune Markers to Determine Safety of Novel Vaginal Products

**DOI:** 10.3389/frph.2022.899277

**Published:** 2022-05-17

**Authors:** Anna-Ursula Happel, Aida Sivro, Lenine Liebenberg, Jo Ann Passmore, Caroline M. Mitchell

**Affiliations:** ^1^Department of Pathology, Institute of Infectious Disease and Molecular Medicine, University of Cape Town, Cape Town, South Africa; ^2^Centre for the AIDS Programme of Research in South Africa (CAPRISA), Durban, South Africa; ^3^Department of Medical Microbiology, University of KwaZulu-Natal, Durban, South Africa; ^4^National Health Laboratory Service, Cape Town, South Africa; ^5^Ragon Institute of MGH, MIT and Harvard, Cambridge, MA, United States; ^6^Harvard Medical School, Boston, MA, United States; ^7^Department of Obstetrics and Gynecology, Massachusetts General Hospital, Boston, MA, United States

**Keywords:** female genital tract, live biotherapeutics, product development, safety, cytokines

## Abstract

Several soluble cytokines have been associated with microbicide-induced cervicovaginal inflammation, non-optimal vaginal microbiota, and risk of HIV acquisition. Many of these biomarkers are used in preclinical assays to estimate the safety of vaginally applied products. However, there are currently no validated biomarkers to evaluate the safety of novel vaginal products in clinical trials. This hinders the rapid and rational selection of novel products being tested in first-in-human trials. We reviewed available literature to assess how best to select and measure soluble immune markers to determine product safety in first in human clinical trials of novel vaginal products.

## Introduction

Vaginally delivered products are used or are in development for contraception, sexual lubrication, prevention of sexually transmitted infections such as human immunodeficiency virus (HIV) and both treatment and prevention of vaginal infections. Currently, preclinical testing of novel products includes safety assessment in the laboratory using cell lines, cervicovaginal tissue or explant models, and small animal models to identify products with significant cytotoxic effects. While these may suffice for basic toxicity assays, humans are the only species in which a vaginal microbiota low in diversity and dominated by *Lactobacillus* species is commonly identified (1, 2). Thus, these models offer only a poor approximation of *in vivo* conditions.

The most well-described consideration for vaginal products is avoidance of hypertonic formulations which are cytotoxic, increase epithelial cell shedding and decrease epithelial integrity (3–5). Inflammation in the human female genital tract, regardless of the cause or presence of symptoms, creates an environment associated with a range of adverse health outcomes, including increased risk of HIV acquisition (6). Any new product should ensure no off-target, pro-inflammatory effects, however optimal safety biomarkers to ensure this are not well established. Currently, many studies of vaginal products measure clinical signs and symptoms. However, vaginal fluid biomarkers are likely more appropriate for this assessment, as there are often no overt clinical signs of inflammation in people at higher risk. The vaginal microbicide field made progress in characterizing markers suggestive of inflammation and mucosal damage, but a threshold for a “danger” signal has not been established (7–10).

Vaginally applied live biotherapeutic products to promote a *Lactobacillus*-dominant microbiota are in varying stages of development. The lack of an animal model for the human vagina means that few products have robust preclinical data describing their impact on inflammation in the human female genital tract in the context of existing microbial communities. In this review, we will address the following questions: Which soluble immune markers should the field focus on when assessing genital inflammation during first in human trials of novel vaginally applied products? How should these be measured, and what signals reflect an increased risk for adverse outcomes-or conversely, indicate the most promising intervention to reduce the risk for adverse outcomes?

## The Cautionary Tale of Nonoxynol-9

The initial realization that a product presumed to be safe and protective could have an unforeseen impact on risk for viral infections occurred with the nonionic surfactant nonoxynol-9 (nonylphenoxypolyethoxyethanol; N-9), which was used as the active component of spermicides for almost 50 years. Early *in vitro* studies of N-9 demonstrated broad-spectrum activity against a number of STIs, including *Chlamydia trachomatis, Neisseria gonorrhoeae*, Herpes simplex virus (HSV*)-2* and HIV (11–18). The *in vitro* data and the widespread use of N-9 as a contraceptive lead to development of this compound as a topical microbicide. Contrary to expectations, the final phase 2/3 clinical trial of N-9 vaginal gel showed an almost 2-fold greater increase in HIV acquisition with high-frequency use of N-9 (19), which was linked to an increase in genital mucosal inflammation.

The strength of the N-9 mediated induction of soluble pro-inflammatory markers was shown to be dependent on dosing and length of exposure (20, 21). A single application of N-9 caused a significant increase in Macrophage Inflammatory Protein (MIP)-1β and RANTES (Regulated upon Activation, Normal T Cell Expressed and Presumably Secreted) by 12h in a mouse model (20). However, in humans, a single application of N-9 did not result in detectable increases in any of the pro-inflammatory markers measured, including interleukin (IL)-1α, IL-1β, IL-8, soluble tumor necrosis factor receptor (sTNF-R)I and sTNF-RII, or IL-1 receptor antagonist (RA). Repeated dosing (3 daily exposures) was needed to observe a significant increase of several markers, including IL-1α, IL-1β, IL-6, IL-8 and MIP-1β at varying times after the last application (12–60 h) (21). Prolonged use of N-9 was associated with increased IL-1β in cervicovaginal secretions and a subsequent increase in IL-1-mediated NFkB activation, resulting in chemokine-induced recruitment of immune cells (21).

Compound-induced damage to the mucosa generally begins with the production of IL-1 and other inflammatory cytokines by damaged epithelial cells. This is followed by NF-kB and AP-1-mediated release of pro-inflammatory cytokines [including IL-1, IL-6, and TNF-a] and chemokines [including IL-8, IL-10, and macrophage inflammatory protein (MIP)-3]. The inflammatory cascade results in the induction of endothelial vascular adhesion molecules, increase in lymphocyte trafficking markers, and the overall immune activation and infiltration of immune cells in the female genital tract (22). This suggests that these cytokines (IL-1, IL-6, and TNF-α), released early during the inflammatory cascade, might be good soluble immune markers to assess a ‘danger signal' during novel product testing in humans.

## The Effects of Endogenous Microbiota

Another source of data to define safety biomarkers are studies of mucosal responses to the vaginal microbiota. Diverse, non-*Lactobacillus* dominant vaginal microbial communities have been associated with epithelial barrier damage, in part through increases in matrix metalloproteinases (MMPs) (23) and apoptosis of vaginal epithelial cells through caspase-3 activation (24–26). Whether by culture, Gram stain or 16S rRNA gene sequencing, women with low abundance of diverse microbes and high abundance of vaginal lactobacilli–such as *L. crispatus*–have lower risk for these adverse outcomes (27–29).

In cross-sectional studies, women with bacterial vaginosis (BV) and/or more diverse, non-*Lactobacillus* dominant communities consistently had higher concentrations of IL-1β and IL-1α than women without BV, with consistently decreased IP-10 concentrations. This is true across studies from several geographic locations (Table 1). Other markers show more variability in studies across continents, and from different decades of analysis, including IL-6, IL-8, IL-10, TNF-α (Table 1).

**Table 1:** Associations between vaginal immune markers and microbiota in cross-sectional studies of non-pregnant, HIV-uninfected women.

**Findings**	**Africa**	**Asia**	**Europe**	**North America**	**South America**
Cytokines elevated with BV (Nugent score)	**IL-1α, IL-1β**, TNF-β, IL-6, IL12p70, IL-8	**IL-1β**, HBD2, HD5, IL-10	**IL-1β**	**IL-1α, IL-1β**, IL-8	**IL-1β**, IL-1RA
Cytokines elevated with diverse vaginal microbial communities	**IL-1α**, TNF-α, IL-1β, IFN-γ, IL-10, IL-8, IL12p70, IL-4, FLT-3L			**IL-1α, IL-1β**, IL-10, GM-CSF	
Cytokines decreased with BV (Nugent score)	**IP-10**	IL-4	IL-10	**IP-10**, MIP-3α, RANTES, MIG, MIP-1β, HNP, HBD1, HBD2, SLPI	IL-10
Cytokines decreased with increasing vaginal microbial diversity				**IP-10**, MCP-1, MIG	
Cytokines measured, but not associated with BV	IL-2, MIP-1α, MIP-1β, IL-1RA, RANTES	IL-2, IL-5, IL-6, IFN-γ	IL-1α, IL-5, IL-12	IL-6, TNF-α, IL-1RA	IL-6, IL-8, TNF-α
References	(30–32)	(33–35)	(36, 37)	(38–40)	(41)

*Bold font highlights markers that yielded common results between studies*.

Longitudinal studies among HIV-uninfected, non-pregnant women showed that transition from a *Lactobacillus*-dominant to a non-*Lactobacillus* dominant microbial community is associated with significant increases in IL-1α, IL-1β, TNF-α, and IL12p70 (30, 42). The converse, a transition to a *Lactobacillus-*dominant community is associated with a decrease in IL-1α, IL-1β, IL-2, IL-4, IL-10, IL-18, TRAIL, TNF-α, and an increase in IP-10, MIG, MCP-1, MIP-1α, MIP-3α, and GROα (43–45). In a US study, treatment of BV was associated with a decrease in GM-CSF and IFN-γ, while in a Kenyan study the two markers increased with decreasing Nugent score (43, 44).

These observations from cross-sectional and longitudinal studies suggest that increases in IL-1α, IL-1β and possibly TNF-α and decreasing IP-10 might be suitable candidates to identify a danger signal in clinical trials.

## Markers of vulnerability

Although several soluble immune markers are commonly increased in response to both N-9 and diverse vaginal microbiota, what matters most for the selection of safety biomarkers is whether those markers are also associated with the risk for acquisition of viral infections such as HIV, HSV and human papillomavirus (HPV). There are few longitudinal studies that assess markers prior to viral acquisition, thus data on the association between specific inflammatory markers and the risk for acquisition are limited.

### HIV Acquisition

In the CAPRISA 004 study, which specifically evaluated HIV acquisition as an endpoint, women with the highest levels of at least five of nine markers of mucosal inflammation were at the highest risk for HIV infection. The panel of nine markers included IL-1α, IL-1β, IL-6, TNF-α, IL-8, IP-10, MCP-1, MIP-1α, MIP-1β, and cytokines were measured a median 4.5 months prior to HIV infection (6, 46). In another South African cohort, CAPRISA 002, increased concentrations of several cytokines and chemokines (IL-1β, IL-6, IL-8, and sCD40L) in cervicovaginal lavage (CVL) were also associated with a greater risk of HIV acquisition despite a long period (median ~300 days) between cytokine measurements in CVL specimens and subsequent HIV acquisition (47). In the FRESH cohort that also enrolled women from South Africa, individuals with a *Lactobacillus*-deficient cervicovaginal microbiota produced higher levels of inflammatory cytokines, particularly IL-1α, IL-1β, TNF-α, IFN-γ, IL-10, and IL-8 (30), and had a greater risk of subsequent HIV acquisition compared to women with *L. crispatus-*dominant genital microbiota (29). In Zimbabwean and Ugandan women, higher RANTES and lower secretory leukocyte protease inhibitor (SLPI) levels were associated with subsequent HIV seroconversion, while no associations between IL-1β, IL-6, IL-8, MIP-3α, ICAM-1, VEGF, and IL-1RA levels and later HIV seroconversion were observed (48–50). While there is some heterogeneity between studies and populations included, higher vaginal fluid IL-1α, IL-1β and IL-8 appear to be associated with increased risk of HIV acquisition. Current studies have not yet determined the absolute concentration threshold associated with increased HIV acquisition risk, or how long that concentration needs to persist to do so.

Additional markers of interest in the context of HIV risk may include biomarkers of epithelial barrier integrity such as matrix metalloproteinases, tissue inhibitors of matrix metalloproteinases (51), cell-cell adhesion markers, and/or select pro-inflammatory cytokines or chemokines related to the influx of T cell targets for infection (6, 29, 51). However, there are fewer data to indicate which specific markers would be the best choice to serve as predictive biomarkers.

### Genital HSV Acquisition

Fewer data are available to define markers for HSV risk. One study in which women were evaluated quarterly found that the highest levels of IL-6, SLPI, ICAM-1 and a higher IL-1RA/IL-1β ratio were associated with a significantly lower risk of genital HSV-2 acquisition by the subsequent quarterly visit (52). IFN-γ, and IFN-stimulated genes appear important for protection against infection with HSV (53, 54). As seen with HIV, BV is a risk factor for HSV acquisition (55). Although IFNs are rarely measured in vaginal fluid samples, IFN-γ induced protein (IP-10) is commonly found to be decreased when vaginal microbiota are diverse and lactobacilli are absent (Table 1).

### HPV Acquisition and Persistence

Even fewer data are available to identify markers for risk of infection with HPV. In one longitudinal study, pre-acquisition levels of vaginal immune markers were not significantly different from those of women who never had HPV. However, post-clearance levels of IL-4, -5, -10, -12, and -13, IFN-γ, IFN-α2, MIP-1α, and TNF-α were significantly elevated compared to pre-acquisition or during infection visits (56). In the CAPRISA 004 study, women who cleared HPV infection had a significant increase in 40/48 measured cytokines and chemokines (57) compared to women who remained HPV negative. In this study, 10/48 cytokines measured were significantly elevated in women who acquired HPV compared to those remaining HPV negative: IL-6, IL12p70, MIF, MIG, MIP-1β, SDF-1α, IL-3, VEGF, IFN-γ, IL-13.

## Variability in Measurement of Biomarkers

A major challenge to defining specific levels of concern for representative biomarkers in this field is the wide variety of mucosal sample types, collection methods, assays and sample processing methods that are used to evaluate immune events in the female genital tract (58, 59). The range of sampling methods for collecting cells and secretions from the FGT include CVL, disposable menstrual cups, swabs or sponge collection from the cervix or lateral vaginal wall, and endocervical cytobrushes (60–62). Within each of these collection methods, variability is further compounded by laboratory processing differences including the dilution factor and diluent used in down-stream processing (58, 59, 63–66).

Most CVL and swab collections cannot accurately adjust for dilution factor (60, 63), which can vary quite significantly from participant to participant (67), making the assessment of actual *in vivo* concentrations and comparison across studies difficult (61, 68–70). Additional participant-specific factors, such as vaginal pH, mucus, the presence of blood or semen (71, 72), or sampling device-specific factors, such as flocked swabs vs. Dacron swabs, or choice of phosphate-buffered saline [pH 7.2] vs. saline [pH 5.5] as a sample diluent have also been shown to impact measurement of cytokine concentrations (58, 63, 73). Inter-laboratory and inter-assay reproducibility of cytokine measurements from cervicovaginal samples is a further concern (58, 74). Nonetheless, although absolute concentrations differ, the relative concentrations of the majority of cytokines correlate between methods (61, 62, 68).

Also, when considering how immune biomarkers should be measured in a safety study, it is important to consider that significant biological variation occurs between and within women. Jespers et al. noted that cervicovaginal cytokine concentrations were more variable within women over time than between different women (42). This implies that the natural variation within a participant may be important context for understanding the impact of a novel product.

## Identifying Biomarkers for Studies of Novel Vaginal Products

Clear cutoff values that would allow the use of biomarkers as a safety signal have not been established. The field needs a panel of markers that reflect the risk of adverse outcomes-a challenge given the lack of data available and the variability between cohorts. The strategy utilized by several groups of identifying a panel inflammatory markers, and categorizing high risk individuals as those with the highest levels of a minimum number of these markers (6, 75) works well within a cohort, identifying individuals with the highest risk relative to others. However, this does not work to relate the risk of participants between cohorts, nor over time.

Absolute values may be helpful at times and for some analyses. At least two studies show differences in vaginal fluid immune markers between healthy populations from different continents (76, 77), even after controlling for hormonal contraception and the presence of BV. These types of comparisons can suggest underlying differences between cohorts that may reflect unmeasured variables and may point to important biological considerations. However, as noted above, the variability in methods between studies means that such comparisons cannot always be made.

When measuring the impact of a vaginal product, our goal is to understand how the product itself changes the risk profile of a given participant. In one small study that measured change in vaginal immune markers after treatment with N-9, cellulose sulfate (CS) or hydroxyethylcellulose (HEC) placebo, the absolute change in markers was not different between CS and placebo. In both placebo and CS arms, markers decreased in absolute value during product use, while N9 was associated with a 10^2^–10^3^ pg/ml increase in IL-1α, IL-1β, IL-1RA, MPO and IL-8 (78). Another strategy would be to measure a ratio of post:pre-treatment concentrations. In one study the comparison of the effect of condomless sex with vs. without lubricant did not identify any differences between the groups (75).

## Conclusion

There are several barriers to providing easy answers to the questions of which biomarkers to use in evaluating novel vaginal products, and what threshold values to use when defining “safe.” These barriers include the lack of clear, definitive data on the exact pathways linking mucosal markers and risk for adverse outcomes; differences in vulnerability profiles for different outcomes; and variability of measurements across studies, which makes direct comparisons or aggregation of data challenging. Based on our review of existing data, we have developed a set of suggested guidelines for studies to use to identify safety signals using soluble vaginal fluid markers (Box 1) and would encourage regulators to support the collection of these markers to better identify what ranges are linked to adverse effects of vaginal products.

Box 1 **Suggested guidelines for measuring soluble immune markers to define safety profiles for novel vaginal products.**

1) Several baseline samples should be obtained prior to product administration to estimate longitudinal variation within a given participant.
2) Longitudinal samples should be collected to facilitate calculation of change in soluble immune markers due to product use. Calculating change in concentrations or ratios between pre/post intervention may allow comparisons between cohorts and minimize the impact of between-cohort variability due to sample processing and measurement methods (79).
3) The least dilute sample type, and one which allows quantification of the volume of vaginal fluid collected should be used to facilitate accurate comparison between measurements
4) The specific markers of interest will depend on what safety outcomes are of interest. To assess the impact of a novel product on risk for acquisition of viral STI, we suggest including measurement of IL-1α, IL-1β and IP-10.

## Author Contributions

JP and CM conceived of the review. All authors contributed to literature review and writing.

## Funding

JP and CM are supported by grants from the Bill & Melinda Gates Foundation.

## Conflict of Interest

JP holds a patent for a Method for diagnosing an inflammatory condition in the female genital tract (EP3063542B1). CM receives grant funding from Merck, and has served as a consultant for Scynexis, Inc. The remaining authors declare that the research was conducted in the absence of any commercial or financial relationships that could be construed as a potential conflict of interest.

## Publisher's Note

All claims expressed in this article are solely those of the authors and do not necessarily represent those of their affiliated organizations, or those of the publisher, the editors and the reviewers. Any product that may be evaluated in this article, or claim that may be made by its manufacturer, is not guaranteed or endorsed by the publisher.
